# Regulation of T cell function by microRNA-720

**DOI:** 10.1038/srep12159

**Published:** 2015-07-22

**Authors:** Yu Wang, Zheng Zhang, Dong Ji, Guo-Feng Chen, Xia Feng, Lu-Lu Gong, Jian Guo, Zhi-Wei Li, Cai-Feng Chen, Bin-Bin Zhao, Zhi-Guo Li, Qi-Jing Li, Hui-Ping Yan, Gregory Sempowski, Fu-Sheng Wang, You-Wen He

**Affiliations:** 1 Department of Immunology, Duke University Medical Center, Durham, NC 27710, USA; 2 Beijing 302 Hospital, Beijing 100039, China; 3 Beijing YouAn Hospital, Beijing 100069, China; 4 Key Laboratory of Systems Biology of Pathogens, Ministry of Health, Institute of Pathogen Biology, Chinese Academy of Medical Sciences & Peking Union Medical College, Beijing 100730, China; 5 Department of Biostatistics and Bioinformatics, Duke University Medical Center, Durham, NC 27710, USA; 6 Department of Medicine, Pathology and Human Vaccine Institute, Duke University Medical Center, Durham, NC 27710.

## Abstract

Chronic hepatitis B virus (HBV) infection is a major global health burden. Functional exhaustion and numerical reduction of HBV-specific cytotoxic T lymphocytes (CTLs) in the liver and peripheral blood limit anti-HBV CTL activity in patients with chronic HBV infection (CHB). However, the ongoing anti-HBV CD8^+^ T cell responses in the lymphoid organs are largely unknown due to the infeasibility of obtaining lymphoid organs from CHB patients. Here we demonstrate that the percentage of HBV-specific CD8^+^ T cells is higher in the spleen of CHB patients than that from peripheral blood and liver. Although they do respond to TCR stimulation and produce IFNγ, the cells proliferate poorly. Furthermore, miR-720 expression is upregulated in HBV-specific CD8^+^ T cells. Overexpression of miR-720 in primary human CD8^+^ T cells inhibits TCR stimulation-induced proliferation. We also demonstrate that TGFβ sustains miR-720 upregulation after TCR stimulation, and blood TGFβ levels are associated with the outcome of type I interferon treatment of CHB patients. Thus, therapies targeting miR-720 may help restore impaired immunity in CHB patients.

Cytotoxic T lymphocyte (CTL) activity mediated by antigen-specific CD8^+^ T cells is essential for viral clearance^1^. Acute viral infection activates the host immune system and induces a robust anti-viral T cell response^2^. During chronic viral infection, CTLs are less numerous than during acute infections, and they exhibit functional impairment referred to as T cell exhaustion^3^. T cell exhaustion occurs in many human chronic viral infections, including chronic HBV (CHB)^4^,^5^,^6^. Despite the rapid advances in the characterization and analysis of T cell exhaustion in mouse models^3^,^7^,^8^, the mechanisms underlying T cell exhaustion in CHB patients are still poorly understood. During CHB, the frequencies of HBV-specific CD8^+^ T cells in the liver and the periphery are as low in viremic patients as in non-infected healthy persons^9^,^10^,^11^. Previous studies have suggested that inhibitory receptors such as PD-1 may cause functional impairment of HBV-specific CD8^+^ T cells in chronic HBV infection^12^. These studies focused on the very limited numbers of peripheral and liver-infiltrating antigen-specific CD8^+^ T cells. However, it remains unknown whether the low frequencies of HBV-specific CD8^+^ T cells in the peripheral blood and patient liver are due to impaired proliferation in the secondary lymphoid organs in CHB patients.

MicroRNAs are endogenous RNAs of approximately 22 nucleotides that imprecisely pair with target mRNAs in mammals^13^ and repress gene expression by destabilizing target mRNAs and/or repressing their translation^14^,^15^. Although accumulating evidence highlights the role of microRNAs in the innate and adaptive immune systems^16^, the role of microRNA in regulating immunity and liver pathogenesis during chronic HBV infection has not been reported.

Here, we show that anti-HBV effector CTLs are present in the spleen of CHB patients at a higher frequency compared to that from periphery. The antigen-specific T cells proliferate poorly upon antigen stimulation *in vitro*. Furthermore, we demonstrate that miR-720 regulates TCR-mediated proliferation of primary human CD8^+^ T cells. We also show that TGFβ promotes miR-720 expression after TCR stimulation, and blood TGFβ levels are associated with the outcome of type I interferon treatment of CHB patients. Thus, our findings suggest that miR-720 regulates T cell function during CHB infection.

## Results

### HBV-specific CD8^+^ T cells are present in the spleens of CHB patients

**Figure 1:**
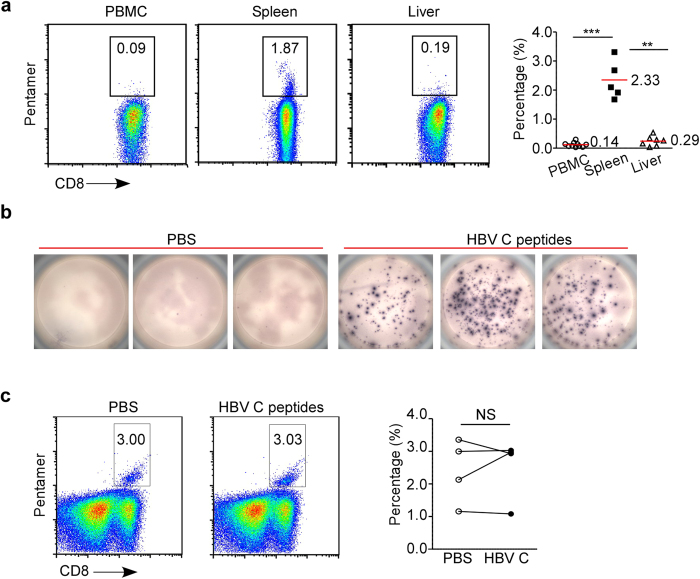
HBV-specific CD8^+^ T Cells in the Spleens of CHB Patients. (**a**) The frequencies of HBV core antigen 18-27 epitope pentamer-positive CD8^+^ T cells among PBMCs, splenocytes, and liver cells from CHB patients were detected by flow cytometry (n = 6, 5 and 6 respectively). Bar indicates mean. (**b**) IFN-γ production by HBV-specific T cells from the spleens of CHB patients was detected by ELISPOT. (c) Frequency of HBV-specific CD8^+^ T cells after 6 days of culture with PBS or HBV core antigen protein (n = 4 and 4). A two-tailed Student’s *t*-test was used to determine significance. **p < 0.01; ***p < 0.001; NS, Not Significant.

Previous studies detected a very rare population of HBV-specific CD8^+^ T cells in the peripheral blood and livers of CHB patients and suggested that impaired clonal expansion and enhanced clonal deletion might account for this observation^9^,^11^,^17^,^18^,^19^,^20^. The anti-HBV T cell response is largely unknown in the lymphoid organ due to the infeasibility of obtaining lymphoid organs from CHB patients. A cohort of CHB patients was used in this study to test the anti-HBV T cell response in the spleen, periphery and liver of CHB patients (Table S1). We analyzed HBV-specific CD8^+^ T cells from the spleens, peripheral blood, and livers of HLA-A2^+^ CHB patients by labeling cells with an HBcAg 18-27 pentamer. As expected, peripheral blood and liver samples from the CHB patients contained a very rare population of HBV-specific CD8^+^ T cells (Fig. 1a). In contrast, the frequency of these cells in the spleen was on average 8-17-fold higher (Fig. 1a), suggesting that antigen-specific CD8^+^ T cells were generated in the secondary lymphoid organs of CHB patients. Start sentence, although HBV-specific splenic CD8^+^ T cells secreted interferon-γ (IFN-γ) in response to stimulation with HBV core antigen peptides (Fig. 1b), they failed to proliferate following six days of antigen stimulation (Fig. 1c). These results demonstrate that splenic HBV-specific CD8^+^ T cells from CHB patients exhibit impaired expansion after antigen stimulation.

### The transcriptional signature of CD8^+^ T cells from CHB patients

**Figure 2:**
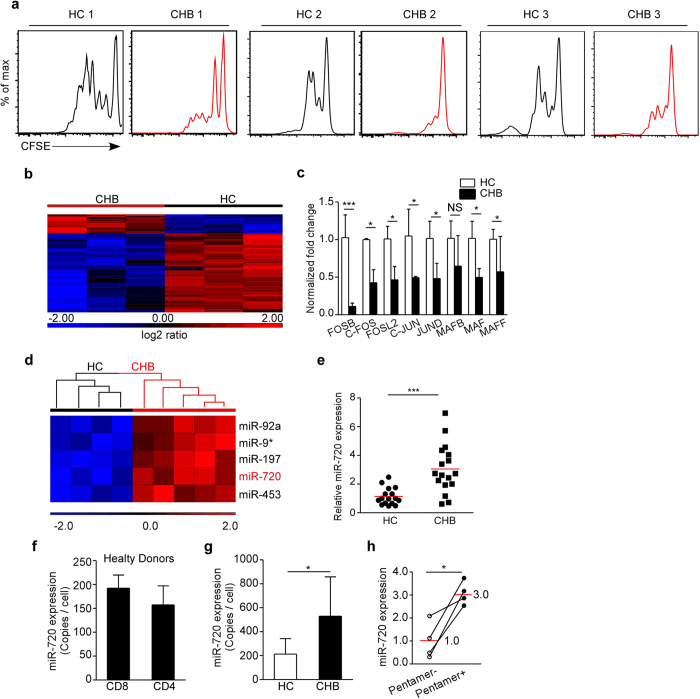
Transcriptional Signature of CD8^+^ T Cells from CHB Patients. (**a**) Freshly isolated PBMCs from different healthy control donors (HC) and CHB patients were stained with CFSE and stimulated for 3-4 days with anti-CD3/CD28. CD8^+^ T cell proliferation was measured by flow cytometry. Data are representative of three independent experiments. (**b**) Heatmap representation of mRNAs with >2-fold differences in CD8^+^ T cells of CHB patients and healthy controls (HC) (n = 3 and 3). (**c**) AP-1 family member mRNA expression in total CD8^+^ T cells of CHB patients and healthy donors (HC) (n = 3 and 3). (**d**) Heatmap representation of microRNAs with >2-fold differences in CD8^+^ T cells of CHB patients and HC (n = 4 and 5). (**e**) miR-720 expression in total CD8^+^ T cells of a different cohort of CHB patients and HC quantitated by qPCR (n = 16 and 17). (**f**) Copy numbers of miR-720 expressed in total CD8^+^ and CD4^+^ T cells from healthy donors. (**g**) Copy numbers of miR-720 expressed in total CD8^+^ T cells from CHB patients and HC (n = 16 and 17). (**h**) miR-720 expression levels in HBV-specific CD8^+^ T cells from the spleens of CHB patients. Shown are expression levels relative to pentamer-negative CD8^+^ T cells from the same patients (n = 4 and 4). Data are represented as mean ± SD in (**c**), (**f**) and (**g**). Bars indicate means in (**e**) and (**h**). A two-tailed Student’s *t*-test was used to determine significance. *p < 0.05; ***p < 0.001; NS, Not Significant.

Our preliminary experiments suggest that the failure to clear HBV infection in CHB patients may be caused by the failed expansion of HBV-specific CD8^+^ T cells; however, the extremely low frequency of HBV-specific CD8^+^ T cells in the peripheral blood and livers of CHB patients has limited studies of the mechanisms underlying HBV-induced T cell exhaustion^9^. Similar to the expansion defect observed in HBV-specific CD8^+^ T cells, TCR-induced proliferation of global CD8^+^ T cells was impaired in a fraction of CHB patients (Fig. 2a)^21^. This finding suggests that examining the molecular regulation of global CD8^+^ T cell function in CHB patients may provide insight into the exhaustion of HBV-specific CD8^+^ T cells. We therefore performed mRNA expression profiling of total CD8^**+**^ T cells purified from peripheral blood mononuclear cells (PBMCs) of healthy donors and CHB patients whose global CD8^+^ T cells displayed proliferation defect. The gene expression pattern of total CD8^+^ T cells from CHB patients differed from that of healthy donors (Fig. 2b, Fig. S1a and b, Table S2). Gene downregulation was the major pattern observed in total CD8^+^ T cells of CHB patients, with ~150 genes downregulated and ~30 genes upregulated compared to total CD8^+^ T cells of healthy donors. Moreover, the expression of AP-1 family members was significantly downregulated (Fig. 2c). Because microRNAs function primarily as negative regulators of gene expression^15^,^16^, we hypothesized that the downregulation of gene expression in total CD8^+^ T cells of CHB patients may be due to the upregulation of microRNAs. Therefore, we performed microRNA expression profiling in these CD8^+^ T cells. Unsupervised hierarchical clustering analysis with a threshold of 2-fold demonstrated that the expression of 5 microRNAs was significantly upregulated in the total CD8^+^ T cells from CHB patients when compared to total CD8^+^ T cells from healthy donors (Fig. 2d). No miRNAs displayed significantly decreased expression in total CD8^+^ T cells from CHB patients (Table S3).

To determine whether the AP-1 family members were potential targets of the upregulated microRNAs, we searched their cDNA sequences for potential microRNA binding sites and found that each AP-1 family member contains at least one potential binding site for miR-720 (Fig. S2). The upregulation of miR-720 expression was further confirmed in total CD8^+^ T cells from another cohort of CHB patients (Fig. 2e). The increased expression of miR-720 was not due to skewing of naïve versus memory CD8^+^ T cell subpopulations in the CHB patients (Fig. S3a and b). Furthermore, the expression levels of miR-720 in the naïve and memory populations of human CD8^+^ T cells of healthy donors were similar (Fig. S3c). We then tested the abundance of miR-720 in human T cells. The copy numbers of miR-720 in normal human CD4^+^ and CD8^+^ T cells were 150-200/cell and increased to ~500/cell in total CD8^+^ T cells of CHB patients (Fig. 2f and g). Moreover, miRNA-720 expression in HBV-specific CD8^+^ T cells of CHB patients was 3-fold higher than in total CD8^+^ T cells from the same patients (Fig. 2h) (approximately 1500 copies/cell).

### Regulation of T cell proliferation by miR-720

**Figure 3:**
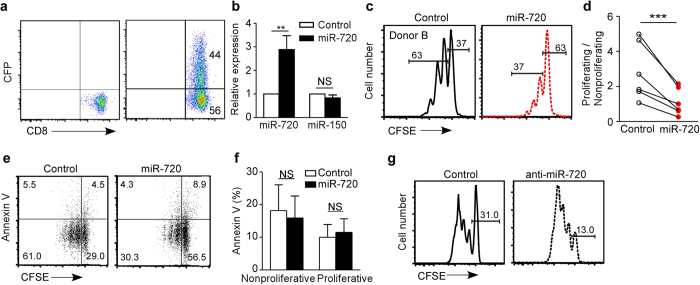
Regulation of CD8^+^ T Cell Proliferation by miR-720. (**a**) Transfection efficiency of pmaxCFP vector containing miR-720 in primary human CD8^+^ T cells. CFP functions as a reporter. (**b**) Overexpression of miR-720 in primary human CD8^+^ T cells using the pmaxCFP-miR vector. qPCR measurement of miR-720 expression levels in sorted pmaxCFP-miR control and pmaxCFP-miR-720-overexpressing CD8^+^ T cells. (**c** and **d**) Proliferation of primary human CD8^+^ T cells overexpressing miR-720. Primary human T cells were electroporated, stimulated with anti-CD3/CD28, and assessed for proliferation. Data are representative of six independent experiments (n = 6 and 6). (**e** and **f**) Flow cytometric analysis of cell death in the proliferating and nonproliferating populations of miR-720-overexpressing and control CD8^+^ T cells. Data are representative of 6 and 4 independent experiments (n = 4 and 4). (**g**) Proliferation of anti-miR-720-treated human primary CD8^+^ T cells. Purified human primary T cells were treated with anti-miR-720 or seed-sequence-mutated antagomirs as described in Methods. The cells were then stained with CFSE and cultured in complete RPMI with anti-CD3/CD28 stimulation for 3-4 days. Cell proliferation was analyzed by flow cytometry. The data are representative of four experiments. Data are represented as mean ± SD in (**b**), and (**f**). A two-tailed paired Student’s *t*-test was used to determine significance. *p <  0.05 with **p <  0.01. NS, Not Significant.

miR-720 shares sequence similarity with tRNA^22^, and tRNA-derived microRNAs also function in B cell^23^. To determine whether miR-720 is a microRNA or a tRNA-derived fragment, we knocked down Dicer in human 293T cells (Fig. S4a) and measured miR-720 expression. miR-720 expression was dramatically decreased upon Dicer knockdown, suggesting that miR-720 is a Dicer-dependent microRNA (Fig. S4b). To test the effect of elevated expression of miR-720 on T cell function, we generated an electroporation-based vector (Fig. S5a) and overexpressed miR-720 in primary unstimulated human T cells at levels similar to those detected in the total CD8^+^ T cells from CHB patients (Fig. 3a and b). Overexpressing miR-720 inhibited human CD8^+^ T cell proliferation (Fig. 3c and d) but did not affect T cell activation (Fig. S5b-d) or survival (Fig. 3e and f). In contrast, silencing of miR-720 using an LNA-based antago-miR-720 enhanced CD8^+^ T cell proliferation by accelerating the cell cycle (Fig. 3g). Taken together, these data suggest that miR-720 may regulate T cell proliferation by targeting cell cycle-related genes.

### miR-720 regulates the expression of cell cycle-related genes

**Figure 4:**
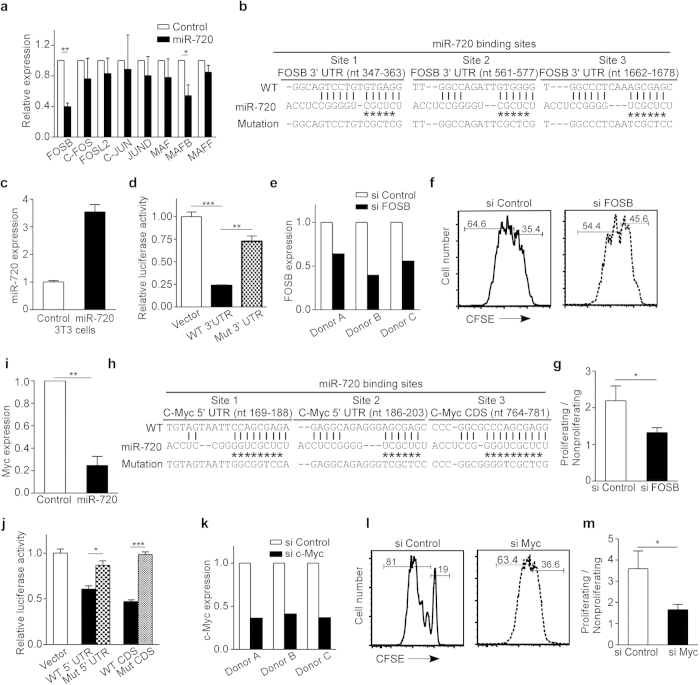
miR-720 Represses Cell Cycle-Related Genes. (**a**) Relative expression levels of AP-1 family genes in miR-720-overexpressing primary hum**a**n CD8+ T cells. Data are representative of four independent experiments (n = 4 and 4). (**b**) Predicted miR-720 targeting sites in the FOSB 3′-UTR. *indicates the seed-sequence mutations. (**c**) Expression levels of miR-720 in stable miR-720-overexpressing 3T3 cell lines. (**d**) Lu**c**iferase activity in cells with miR-720 seed-sequence mutations at the FOSB 3′-UTR. Data are representative of three independent experiments. (**e**) FOSB mRNA expression in FOSB siRNA-silenced human primary CD8+ T cells as determined by qPCR. (**f** and **g**) Proliferation of human CD8^+^ T cells after siRNA-mediated Fosb silencing. Data are representative of four independent experiments (n = 4 and 4). (**h**) Predicted miR-720 targeting sites in the c-Myc 5′-UTR and CDS. * indicates seed-sequence mutations. (**i**) Myc mRNA expression in miR-720-overexpressing primary human CD8^+^ T cells compared to control siRNA-transfected cells. Data are representative of three independent experiments (n = 3 and 3). (**j**) Luciferase activity in cells with miR-720 seed-sequence mutations at the c-Myc 5′-UTR and CDS. Data are representative of three independent experiments (**k**) c-Myc mRNA levels after siRNA silencing in primary CD8^+^ T cells as measured by qPCR. (**l** and **m**) Proliferation of primary human CD8^+^ T cells after siRNA-mediated Myc silencing. Data are representative of four independent experiments (n = 4 and 4). Data are represented as mean ± SD in (a, c, d, g, i, j and m). Two-tailed paired (a, g, i and m) and unpaired (d and j) Student’s *t*-tests were used to determine significance. *p < 0.05; **P < 0.01; ***p < 0.001.

Because the mRNA expression of AP-1 family members was reduced in CD8^+^ T cells from CHB patients (Fig. 2c) and AP-1 family members are well known cell cycle regulators, we next measured the expression of AP-1 family members in miR-720-overexpressing CD8^+^ T cells of healthy donors. Overexpressing miR-720 in primary CD8^+^ T cells decreased the mRNA levels of FOSB and MAFB but not of other AP-1 family members (Fig. 4a). Because MAFB expression was not decreased in the CD8^+^ T cells of CHB patients (Fig. 2b), we next tested whether FOSB is a direct target of miR-720 and examined the role of FOSB in human T cell proliferation. The 3′-untranslated region (UTR) of FOSB contains 3 potential miR-720 target sites (Fig. 4b). To test the effect of miR-720 targeting on FOSB, we generated a stable miR-720-overexpressing 3T3 cell line (Fig. 4c) and confirmed that FOSB is a direct target of miR-720 by performing a luciferase assay (Fig. 4d). To test the role of FOSB in human T cell proliferation, we used an siRNA pool to silence FOSB in primary CD8^+^ T cells from healthy donors (Fig. 4e). The levels of FOSB after silencing were similar to those in miR-720-overexpressing cells (Fig. 4a), and silencing FOSB was sufficient to reduce the proliferation of primary human CD8^+^ (Fig. 4f and g) and CD4^+^ T cells (Fig. S6a).

FOSB silencing partially recapitulated the effect of miR-720 overexpression on T cell proliferation, suggesting that miR-720 may also regulate other cell cycle-related genes. The cell cycle regulator Myc contains multiple miR-720 targeting sites in both the 5′-UTR and the coding region (CDS) (Fig. 4h), and Myc expression is induced by stimulators of cell proliferation in human lymphocytes^24^. We measured Myc mRNA expression in anti-CD3/CD28-activated human T cells overexpressing miR-720. Myc mRNA expression was decreased by 75% in miR-720-overexpressing CD8^+^ T cells when compared to control transfected cells (Fig. 4i). Targeting of Myc by miR-720 was further confirmed by luciferase assay (Fig. 4j). Furthermore, siRNA-mediated silencing of Myc in human T cells (Fig. 4k) resulted in impaired proliferation of CD8^+^ (Fig. 4l and m) and CD4^+^ T cells (Fig. S6b).

Exhausted T cells display impaired cytokine production; however, elevated miR-720 expression in human primary CD8^+^ T cells did not alter their overall cytokine production profile (Fig. S7a), suggesting that miR-720 does not target these specific cytokines during chronic HBV infection. Inhibitory receptor upregulation was observed in exhausted T cells during human chronic viral infection. Elevated miR-720 expression did not enhance the expression of PD-1, LAG3, and CTLA-4 at either early or late stages of T cell activation (Fig. S7b). Taken together, these data suggest that the skewed cytokine production and increased inhibitory receptor expression observed in CHB patients are not due to elevated miR-720 expression.

### TGFβ and TCR signaling promotes miR-720 expression

**Figure 5:**
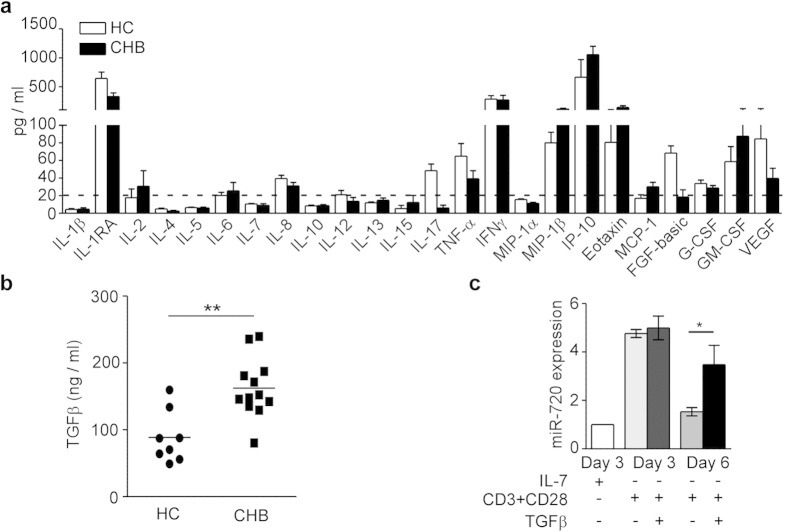
TGFβ and TCR Signaling Promote miR-720 Expression. (**a**) Cytokine profile of plasma from CHB patients (Luminex assay; n = 10 and 12 respectively). (**b**) TGFβ levels in the plasma from CHB patients and healthy control donors (HC) (n = 8 and 12). (**c**) miR-720 expression levels in resting and activated CD8^+^ T cells with or without anti-CD3, anti-CD28, or TGFβ. Data are representative of four independent experiments (n = 4 and 4). Data are represented as mean ± SEM in (**a**) and (**c**). Bar indicates mean in (**b**). Two-tailed unpaired (**b**) and paired (**c**) Student’s *t*-tests were used to determine significan**c**e. *p < 0.05; **P < 0.01.

Chronic HBV infection induces liver damage and systemic inflammation. Inflammatory cytokines may play a role in miR-720 upregulation in total CD8^+^ T cells, and TCR signaling may further enhance miR-720 expression in HBV-specific CD8^+^ T cells (Fig. 2h). We measured cytokine profiles in the plasma of CHB patients and healthy donors. Several cytokines, including IP-10, eotaxin, and TGFβ, were upregulated in the CHB plasma samples (Fig. 5a and b); however, the increases in IP-10 and eotaxin were not statistically significant due to large variations in the healthy controls. To test the roles of cytokines and TCR signaling in miR-720 expression, primary human CD8^+^ T cells were cultured with or without TCR stimulation in the presence of cytokines. TCR stimulation alone dramatically upregulated miR-720 expression on day 3 (Fig. 5c). TGFβ did not induce miR-720 upregulation in resting human CD8^+^ T cells (data not shown); however, TGFβ functioned to maintain the upregulated miR-720 expression induced by TCR stimulation (Fig. 5c). The other two cytokines, IP-10 and eotaxin, had no effect on miR-720 expression (data not shown). These results suggest that persistent TCR stimulation and TGFβ signaling promote miR-720 expression.

### Association of miR-720 and TGFβ levels with treatment outcome

**Figure 6:**
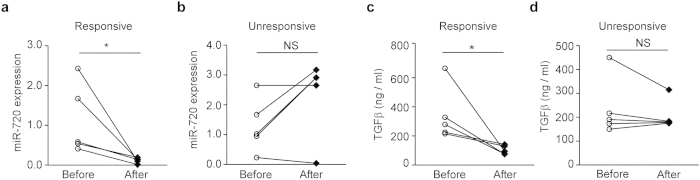
Association of miR-720 and TGFβ Levels with Treatment Outcome. miR-720 level in CD8 T cells from responsive (**a**) and nonresponsive (**b**) patients after IFN-α treatment (n = 5 and 5). “Before” indicates day 0, when the patients were recruited and had received no prior anti-viral treatment. “After” in the Responsive Group indicates the time point of HBeAg to anti-e antibody seroconversion. Viral loads were undetectable for an average period of 8.4 months. “After” in the Unresponsive Group indicates that at the end of the 24 months of IFN-α treatment, these patients remained anti-HBeAg antibody-negative. Plasma TGFβ levels from responsive (**c**) and nonresponsive (**d**) patients after IFN-α treatment as indicated in (**a**) (n = 5 and 5). Two-tailed paired Student’s *t*-tests were used to determine significance. *p < 0.05; NS, Not Significant.

We next examined whether miR-720 levels in total CD8^+^ T cells were correlated with the treatment outcome of CHB patients who underwent interferon alpha (IFN-α) anti-viral therapy^25^. The expression of miR-720 in total CD8^+^ T cells decreased after successful treatment in a group of IFN-α-responsive HBeAg seroconverted CHB patients; however, miR-720 expression was unchanged or even further increased in total CD8^+^ T cells from non-responsive CHB patients (Fig. 6a and b), suggesting that a reduction in miR-720 expression is associated with IFN-α-mediated restoration of anti-HBV T cell immunity. Moreover, plasma TGFβ levels were decreased to normal levels in IFN-α-responsive HBeAg seroconverted CHB patients (Fig. 6c), whereas plasma TGFβ levels were unchanged in treatment non-responsive CHB patients (Fig. 6d).

## Discussion

Chronic HBV infection afflicts 350 million people worldwide and causes 1 million deaths annually^26^. Effective control of chronic HBV infection likely requires the generation of effector T cells in the secondary lymphoid organs and a robust CTL response in the liver. Due to practical reasons, previous studies mainly focused on studying HBV-specific CD8^+^ T cells from the peripheral blood and livers of CHB patients. These studies suggested several possible causes for the paucity of HBV-specific CD8^+^ T cells in the peripheral blood and livers of CHB patients, including impaired proliferation and enhanced apoptosis^9^,^11^,^17^,^18^. However, these studies did not address the ongoing anti-HBV T cell response in the lymphoid organ and the signature of these effector T cells. Addressing this question is critical to understand the failure of the host anti-viral response in chronic HBV infection.

By studying CD8^+^ T cells from the spleens, peripheral blood, and livers of the same group of CHB patients, we have made several findings. First, we found that HBV-specific CD8^+^ T cells are generated in the spleens of CHB patients. However, they have impaired capacity to expand after stimulation. Second, we have identified miR-720 as a key regulator of CD8^+^ T cell proliferation by targeting the expression of the cell cycle regulators FOSB and c-Myc. Third, we have identified TCR signaling and TGFβ as stimuli of miR-720 expression in T lymphocytes and demonstrated that the expression of miR-720 in CD8^+^ T cells is strongly correlated with the treatment outcome of CHB patients. Taken together, our results suggest that upregulation of miR-720 in CD8^+^ T cells may play an important role in the development of chronic HBV infection: it inhibits antigen-specific CD8^+^ T cell expansion in secondary lymphoid organs, leading to insufficient antigen-specific CD8^+^ T cells migrating to the liver. The effects of miR-720 on the proliferation of HBV-specific and total CD8^+^ T cells during HBV infection may result in the transition from acute hepatitis B to persistent infection and hepatitis.

Our results suggest that upregulation of miR-720 in HBV-specific T lymphocytes plays a critical role in host immunity during chronic HBV infection. Our data are consistent with a model in which HBV-specific CD8^+^ T cells are activated in the spleen by HBV antigens, resulting in miR-720 upregulation. The elevated miR-720 expression is further sustained by high levels of TGFβ, thus preventing the generation of sufficient antigen-specific effector T cells.

Our results indicate that miR-720 is an important regulator of T cell proliferation. Primary T cells from healthy donors contain ~200 copies of miR-720 per cell, whereas total CD8^+^ T cells and HBV-specific CD8^+^ T cells from CHB patients contain ~500 and ~1500 copies of miR-720, respectively. When we overexpressed miR-720 in normal T cells at a level of ~500 copies per cell, T cell proliferation was impaired. Furthermore, treatment of primary T cells with miR-720 antagomir promotes their entry into the cell cycle. miR-720 regulates T cell proliferation by targeting two known cell cycle regulators, AP-1 and Myc. A previous study suggests that cooperation of NFAT and AP-1 is important for T cell activation and proliferation^27^. Our microarray data show that NFAT expression is intact; however, AP-1 expression is reduced. Moreover, FOSB silencing inhibits the proliferation of human primary T cells. These data support the idea that miR-720 regulates T cell exhaustion partially by altering the NFAT:AP-1 balance. Importantly, c-Myc expression is also suppressed by miR-720 during T cell proliferation. In T cells, c-Myc is induced upon T cell activation and drives T cells into the cell cycle^28^. Thus, miR-720 regulates the cell cycle at both early and later stages during T cell activation.

Our data suggest that the elevated expression of miR-720 in HBV-specific CD8^+^ T cells may be caused by a combined signal from TCR engagement and TGFβ signaling. Both viral antigens and TGFβ have been implicated in CD8^+^ T cell functional exhaustion in chronic viral infections^19^,^29^,^30^. In mouse LCMV chronic infection, TGFβ signaling mediates virus-specific CD8^+^ T cell deletion and viral persistence. In chronic HBV infection in humans, TGFβ1 signaling downregulates activating NK receptor expression and may contribute to HBV persistence^31^. Our results provide important insights into the roles of viral antigen and TGFβ in causing T cell exhaustion: these signals also upregulate miR-720 expression, leading to repressed expression of FOSB, and c-Myc and impaired proliferation of effector CD8^+^ T cells.

Clinically, nucleotide analogues and IFN-α are used to treat HBV infection^25^. Nucleotide analogues can efficiently inhibit viral replication; however, they also induce HBV DNA mutation, drug resistance, and poor HBV e antigen (HBeAg) seroconversion^26^. IFN-α exerts its anti-HBV effect through specific and non-specific antiviral immune responses^32^. Despite the comprehensive application of IFN-α treatment to chronic HBV infection, the outcome of this treatment is unpredictable. Our data indicate that miR-720 expression and plasma TGFβ levels are associated with the outcome of IFN-α treatment and suggest that together, miR-720 expression in CD8^+^ T cells and plasma TGFβ levels could be used as biomarkers for IFN-α treatment outcome prediction in CHB patients. In summary, our findings provide evidence that miR-720 plays a key role in HBV-specific T cell exhaustion during chronic HBV infection. Hence, targeting miR-720 may be a novel strategy to reverse T cell exhaustion and alleviate liver damage.

## Methods

### Human Subjects

All human patients and healthy donor controls were recruited at Beijing 302 Hospital and Beijing YouAn Hospital in accordance with the IRB-approved study “The key role of microRNA during chronic HBV infection and T cell tolerance”; Protocol # 2013035D and JYL2010-26. Tissues and peripheral blood samples were collected after receiving the patient’s informed consent. PBMCs were obtained from HBeAg-positive patients infected with HBV for at least 5 years with viral loads >10^6^ IU/ml. These patients were treatment-naive. Non-hepatitis control liver specimens were obtained during liver haemangioma surgeries from patients who were HBV-, HCV-, and HIV-negative. CHB liver biopsies were obtained from patients who developed chronic HBV and had viral loads >10^5^ IU/ml. Spleen specimens were obtained during splenectomies performed on portal hypertension patients with chronic HBV infection. The criteria for IFN-α treatment cohorts were that the patients did not receive any anti-viral treatment and viral loads were >10^6^ before treatment. Blood samples were drawn at different time points during a 2-year course of treatment. Healthy donors providing PBMCs for transfection studies were recruited at Duke University Medical Center in accordance with the IRB-approved study “Virologic Basis for Specific Immune Defects in AIDS”; Protocol # 00004020. More detailed materials and methods are presented in the supplementary Materials and Methods.

## Additional Information

**How to cite this article**: Wang, Y. *et al.* Regulation of T cell function by microRNA-720. *Sci. Rep.* **5**, 12159; doi: 10.1038/srep12159 (2015).

## Supplementary Material

Supplementary Information

Supplementary Table S2

Supplementary Table S3
