# Supplementary material for: Regulation of T cell function by microRNA-720
Source: Sci Rep. 2015 Jul 22;5:12159. doi: 10.1038/srep12159 (PMC4510490; doi:10.1038/srep12159)
Supplement: Supplementary Information [file srep12159-s1.doc]

Regulation of T cell function by microRNA-720

Yu Wang^1^, Zheng Zhang^2*^, Dong Ji^2*^, Guo-Feng Chen^2^, Xia Feng^3^, Lu-Lu Gong^4^, Jian Guo^1^, Zhi-Wei Li^2^, Cai-Feng Chen^4^, Bin-Bin Zhao^4^, Zhi-Guo Li^5^, Qi-Jing Li^1^, Hui-Ping Yan^3^, Gregory Sempowski^6^, Fu-Sheng Wang^2^ & You-Wen He^1^

Supplementary Material

Supplementary methods

**Human Leukocyte Isolation and Flow Cytometric Analysis.** Human PBMCs were enriched using Ficoll (GE Healthcare, Pittsburgh, PA). Splenocytes were extracted by mechanical disruption of human spleens, filtered through 100-μm stainless steel mesh, and enriched using Ficoll twice. Liver-infiltrating leukocytes were extracted by mechanical disruption. Anti-CD3, -CD4, -CD8 -CD45RA, and -CD45RO antibodies were purchased from Biolegend (San Diego, CA). An HBV core antigen 18-27 peptide-loaded HLA-A2 specific pentamer was purchased from ProImmune (Oxford, OX4 4GA, UK). For the HBV-specific CD8^+^ T cell staining, PE-labeled HBcAg 18-27-loaded pentamer was incubated with PBMCs, splenocytes, and liver-infiltrating leukocytes for 10 minutes at 22°C. The cells were stained with CD3, CD4, and CD8 antibodies and analyzed using a BD FACSCaliber or FACSCanto II flow cytometer (BD Biosciences, San Jose, California) with FlowJo software (Tree Star, Ashland, OR).

**ELISPOT Assays.** Assays were carried out in 96-well MultiScreen filter plates (Millipore, Billerica, MA) coated with 10 µg/ml of anti- IFN-γ monoclonal antibody (Mabtech, Stockholm, Sweden). Splenocytes were added at a density of 5 x 10^4^ cells/well and then stimulated with HBV core antigen peptides (2 µg/ml final concentration) for 16 h at 37°C in the presence of 5% CO_2_. Cells cultured in RPMI alone and with CD3 stimulation served as negative and positive controls, respectively. ELISPOT assays were performed according to the manufacturer’s instructions.

Human CD8^+^ T Cell Isolation and RNA Extraction. Fifteen milliliters of blood was drawn from each CHB patient and healthy donor. PBMCs were enriched using Ficoll, and CD8^+^ T cells were purified using positive selection beads to a purity of >95% (Miltenyi Biotec, Auburn, CA). Total RNA was extracted using a mirVana miRNA isolation kit (Life Technologies, Carlsbad, CA).

mRNA Gene Chip Array and Data Analysis. Total RNAs were assessed for quality with an Agilent 2100 Bioanalyzer G2939A (Agilent Technologies, Santa Clara, CA) and a Nanodrop 8000 spectrophotometer (Thermo Scientific/Nanodrop, Wilmington, DE). Hybridization targets were prepared with MessageAmp Premier RNA Amplification Kit (Applied Biosystems/Ambion, Austin, TX) from total RNA, hybridized to AffGeneChip Human Genome U133 Plus 2.0 Arrays in an Affymetrix GeneChip hybridization oven 645, washed in an Affymetrix GeneChip Fluidics Station 450, and scanned with an Affymetrix GeneChip® Scanner 7G according to standard Affymetrix GeneChip® Hybridization, Wash, and Stain protocols (Affymetrix, Santa Clara, CA). Raw intensities from the CEL files were analyzed using Partek Genomics Suite (Partek Incorporated, St. Louis, MO) with standard background correction to generate an RMA (robust multi-array average intensity) on a log2 scale for each probe set. Probe sets were filtered for “present” detection in two or more arrays, and the interquartile intensity range was >0.5. The filtered RMA intensities were then analyzed for differential expression using the advanced analysis of variance (ANOVA), and differentially expressed genes were generated by FDR-adjusted P values using the Benjamini-Hochberg method.

microRNA Realtime PCR Array and Data Analysis. A Poly (A) Polymerase Tailing Kit (****Epicentre Biotechnologies,**** ****Madison, WI****) was used to add poly (A) to the 3’ end of total RNA. The poly (A)-tailed total RNA was then reverse transcribed to cDNA with a universal primer using Superscript III (Life Technologies, Carlsbad, CA). Realtime PCR arrays were performed using 350 microRNA-specific primers in 96-well plates. Original microarray data were normalized to the mean Ct and log2 transferred, and relative fold changes were calculated using the delta-delta Ct algorithm. Differentially expressed microRNAs were analyzed by ANOVA.

microRNA Copy Number Measurement. To determine the abundance of miR-720 in human T cells, purified CD4^+^ and CD8^+^ T cells were spiked with a chemically synthesized miR-720 at fixed copy numbers/cell (0, 100, 200, 400, 800, and 1600 copies/cell). Total RNAs were extracted using a mirVana kit (Life Technologies). Quantitative realtime PCR was performed, and U6 was used as loading control. miR-720 copy numbers were calculated based on the Ct difference between the sample spiked with 0 copies/cell and the other samples spiked with other concentrations of miR-720. For example, finding that the sample spiked with 200 copies/cell displayed a Ct value that was one cycle lower than the sample spiked with 0 copies/cell would indicate that the endogenous miR-720 copy number is 200 copies/cell.

**RT-PCR, RNA Silencing, microRNA Overexpression and Antagomir.** Quantitative realtime PCR was performed using a SYBR green-based assay (Applied Biosystems). For mRNA expression, 18s rRNA was used for normalization across samples. For microRNA expression, the small nuclear RNA U6 was used for normalization.

**microRNA Target Prediction.** MicroRNA targets were predicted using the Targetscan algorithm, which considers site conservation, and the RNA22 microRNA target detection algorithm, which does not consider site conservation.

Generation of microRNA Expression Vector. CFP was generated from GFP using site-directed mutagenesis as described and cloned into pMax vector downstream of the CMV-chimeric intron, which can significantly increase transgene expression. Sal I and EcoR I sites were generated between the donor site and the branch site in the chimeric intron for microRNA cloning using site-directed mutagenesis. This vector efficiently expresses microRNAs in quiescent and activated primary human T cells.

**microRNA Overexpression and Gene Knockdown in Primary Human PBMCs.** PBMCs from healthy donors were diluted with two volumes of PBS containing 0.5% BSA (wash buffer) and separated by Ficoll at 750 x g for 20 min. The cells were then washed 3 times according to the manufacturer’s instructions. PBMCs were suspended in human T cell nucleofector solution. Ten million PBMCs were mixed with 5 µg pmaxCFP-miR control or pmaxCFP-miR-720 plasmids and electroporated using an AMAXA Nucleofector (Lonza, Allendale, NJ). For gene silencing, 1x10^7^ PBMCs were mixed with control siRNA, a Fosb siRNA pool, or a c-Myc siRNA pool (Thermo Scientific, Pittsburgh, PA) and electroporated using an AMAXA Nucleofector. The cells were cultured with nucleofector solution for 4-6 hours; the medium was then replaced with fresh complete RPMI medium, and the cells were cultured overnight.

**Antagomir Treatment.** miR-720 and seed-sequence mutant control antagomirs were synthesized by Thermo Scientific. Human T cells were purified using a negative selection kit (STEMCELL Technologies, Vancouver, BC, Canada). Ten million T cells were resuspended in 50 µl serum-free medium (Life Technologies). The miR-720 or seed-sequence mutant antigomirs (50 µg/ml) were added to the cells in the presence of 2 ng/ml IL-7 for 30 min at 37°C. The cells were then stained with CFSE and cultured for 3-4 days with anti-CD3/CD28 for proliferation assays.

**CFSE Labeling and Cell Cycle Analysis.** PBMCs were labeled with CFSE as described. To measure cell proliferation, 3x10^5^ PBMCs were seeded into one well of a 96-well plate and stimulated with 1 μg /ml anti-CD3/CD28 for 3-5 days. The cells were stained with anti-CD8 antibody and annexin V before flow cytometry analysis.

**Luciferase Assays.** 3T3 cells were infected with a miR-720-bearing MSCV virus, and stable lines were selected with puromycin. Wild type and mutant Fosb 3’UTR, c-Myc 5’UTR and CDS were cloned into the pmirGLO vector (Promega, Madison, WI). miR-720-overexpressing 3T3 cells were transfected with 0.5 µg of the pmirGLO plasmid in 24-well plates using Lipofectamine 2000 (Invitrogen). Firefly and renilla luciferase activities were measured in cell lysates 48 hours post-transfection using the Dual-Luciferase Reporter Assay System (Promega). Relative firefly luciferase activity was normalized to renilla luciferase activity.

**Luminex Assay.** Plasma from healthy controls and CHB patients were diluted at 1:5. Luminex assay was were performed according to the manufacturer’s instructions using human 30-plex panel (Invitrogen).

Statistical Analysis. **Two tailed Student’s *t*-test (Paired and Unpaired) was performed using Prism software (Graphpad, La Jolla, CA).**

Quantitative realtime PCR primer sequences:

FOSB (NM_001114171)

Forward: 5' CTTCCGATCCCCTGAACTC 3'

Reverse: 5' CACTTTGTCTCTTCCCCTCTC 3'

C-FOS (NM_005252)

Forward: 5' TTGTGAAGACCATGACAGGAG 3'

Reverse: 5' CCATCTTATTCCTTTCCCTTCGG 3'

FOSL2 (NM_005253)

Forward: 5' CGGGAGCTGACAGAGAAG 3'

Reverse: 5' GGGCTAATCTTGCACACTGG 3'

JUN (NM_002228)

Forward: 5' ACTTTCCCTGTCAAAGGCTC 3'

Reverse: 5' GCAGTCATAGAACAGTCCGTC 3'

JUND (NM_005354)

Forward: 5' GGAGGCGGAGGATGGAAACA 3'

Reverse: 5' TCACTCAGGCTCAGCGTCAG 3'

MAFB (NM_005461)

Forward: 5' CGAGCAACTACCAGCAGATGAAC 3'

Reverse: 5' GACGCTTGGTGATGATGGTGATG 3'

MAF (NM_001031804)

Forward: 5' TGGCTGGCTGTAAGAGAAGGTTAA 3'

Reverse: 5' AGTGGGCTCAGTTCTGTAATTGGA 3'

MAFF (NM_001161572)

Forward: 5' GGCACCTTCTGCAAACATG 3'

Reverse: 5' CAGATGCCGGTTCAGCTC 3'

MYC (NM_002467)

Forward: 5' GGACCCGCTTCTCTGAAAG 3'

Reverse: 5' GTCGAGGTCATAGTTCCTGTTG 3'

18S rRNA (NR_003286)

Forward: 5' TCAACTTTCGATGGTAGTCGCCGT 3'

Reverse: 5' TCCTTGGATGTGGTAGCCGTTTCT 3'

miR-720 cloning primers:

Short human-720

Forward: 5' CCAACTCGAG TCTGGAAGACGGTCACTCTTCG 3'

Reverse: 5' CCAAGAATTC CTAAACCCCCCCTCCCCTTC 3'

microRNA target region cloning primers:

FOSB

Forward: 5' ACTCTTTAGACACACAAAACAAACAAAC 3'

Reverse: 5' GGCAACAGTGCAGAACCAAGGG 3'

MYC 5’UTR

Forward: 5' ATGCGTCGACCCCCGAGCTGTGCTGCT 3'

Reverse: 5' ATGCGAATTCGACCGCTGGCTGGGGGATCA 3’

MYC CDS

Forward: 5' ATCG CTCGAGTGGAAAACCAGCAGCCTCCCG 3'

Reverse: 5' ATGC GAATTCGCCGTCGTTGTCTCCCCGAA 3'

**Supplementary Figures**


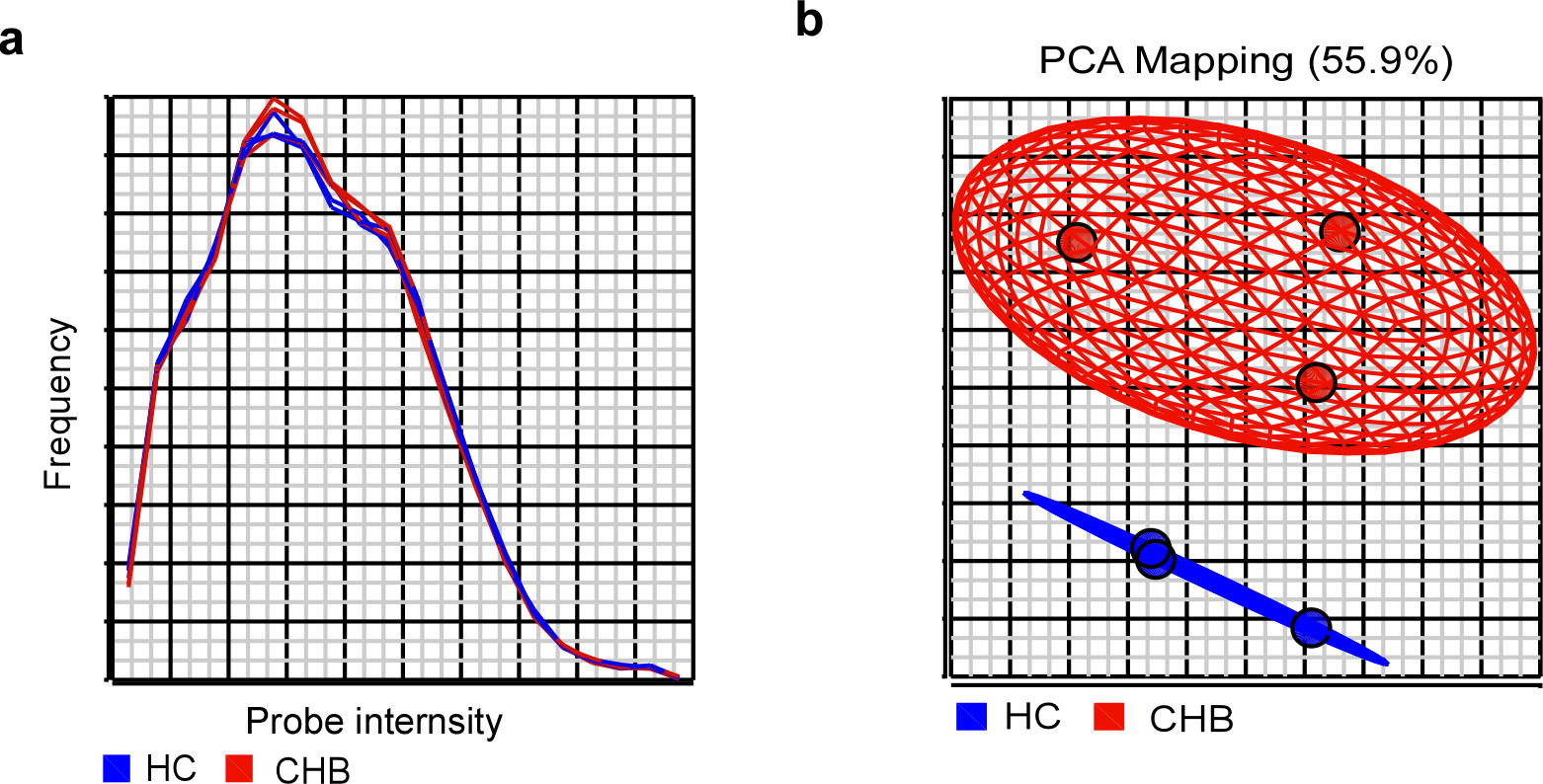


**Figure S1 | Transcriptional profiles of proliferation-defective total CD8^+^ T cells from CHB patients**. Freshly isolated PBMCs from different healthy control donors (HC) and CHB patients were enriched for CD8 T cells using negative selection beads. Microarray was done as described in Methods. (a) Similar Probe intensity distribution indicating the comparability between HC and CHB microarray data. Briefly, the human 133A array contains 45,000 probes representing more than 39,000 transcripts. The X-axis represents the intensity of different probes, and the Y-axis represents the frequency of probes at each probe intensity. Similar probe distributions indicate well-controlled microarray quality. (b*)* As shown in the principal component analysis (PCA), the CHB group can be separated from the HC group by the first PCA, which captured 55.9% of the overall variance. A PCA Mapping score of 55.9% means that CHB patients can be separated from the HC group based on 55.9% of the total variance (39,000 transcripts).


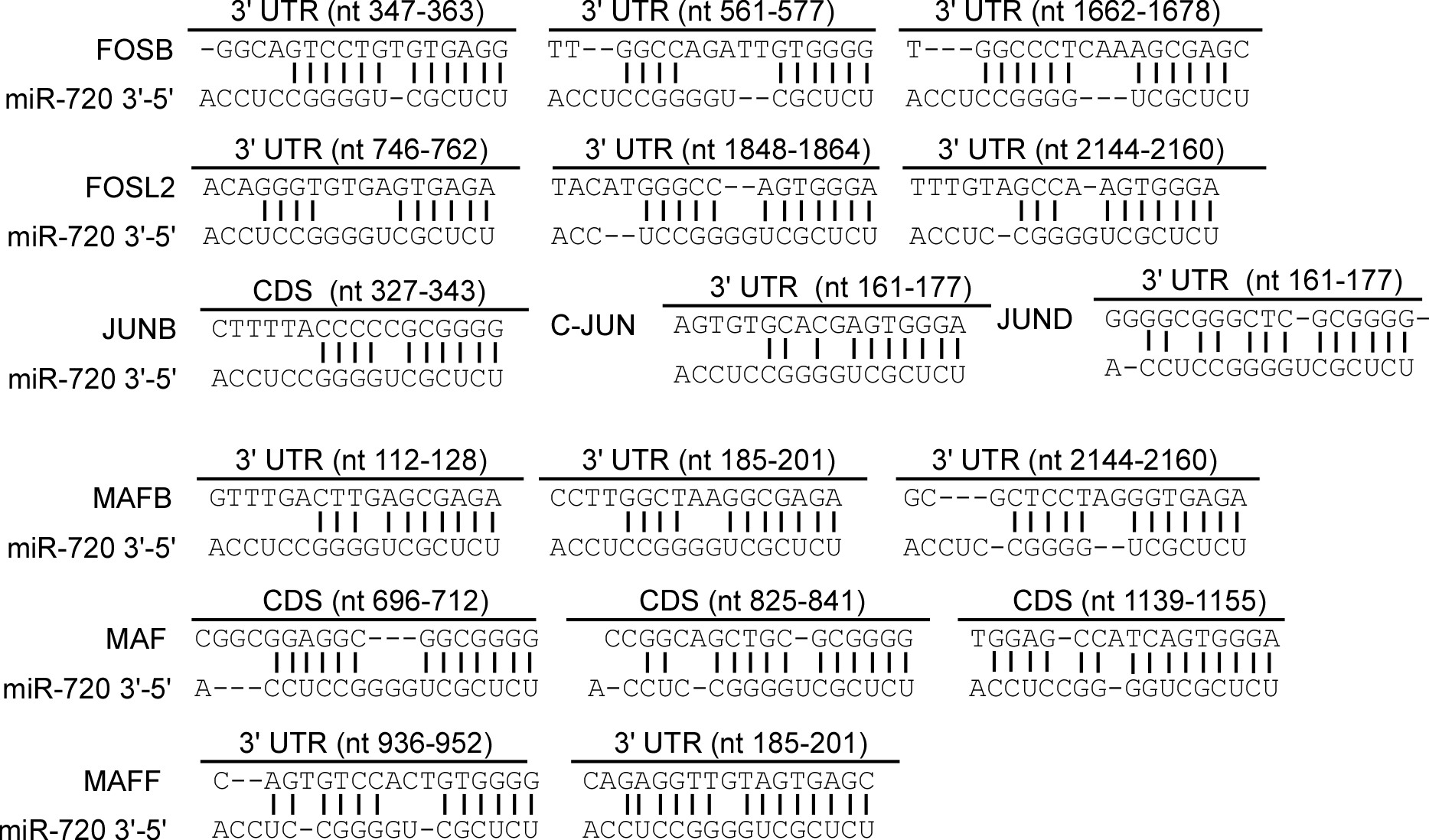


**Figure S2 | Predicted target genes and binding sites of miR-720. Multiple algorithms were used to predict miR-720 targets as described in Methods**. Predicted miR-720 binding sites in the AP-1 family genes.


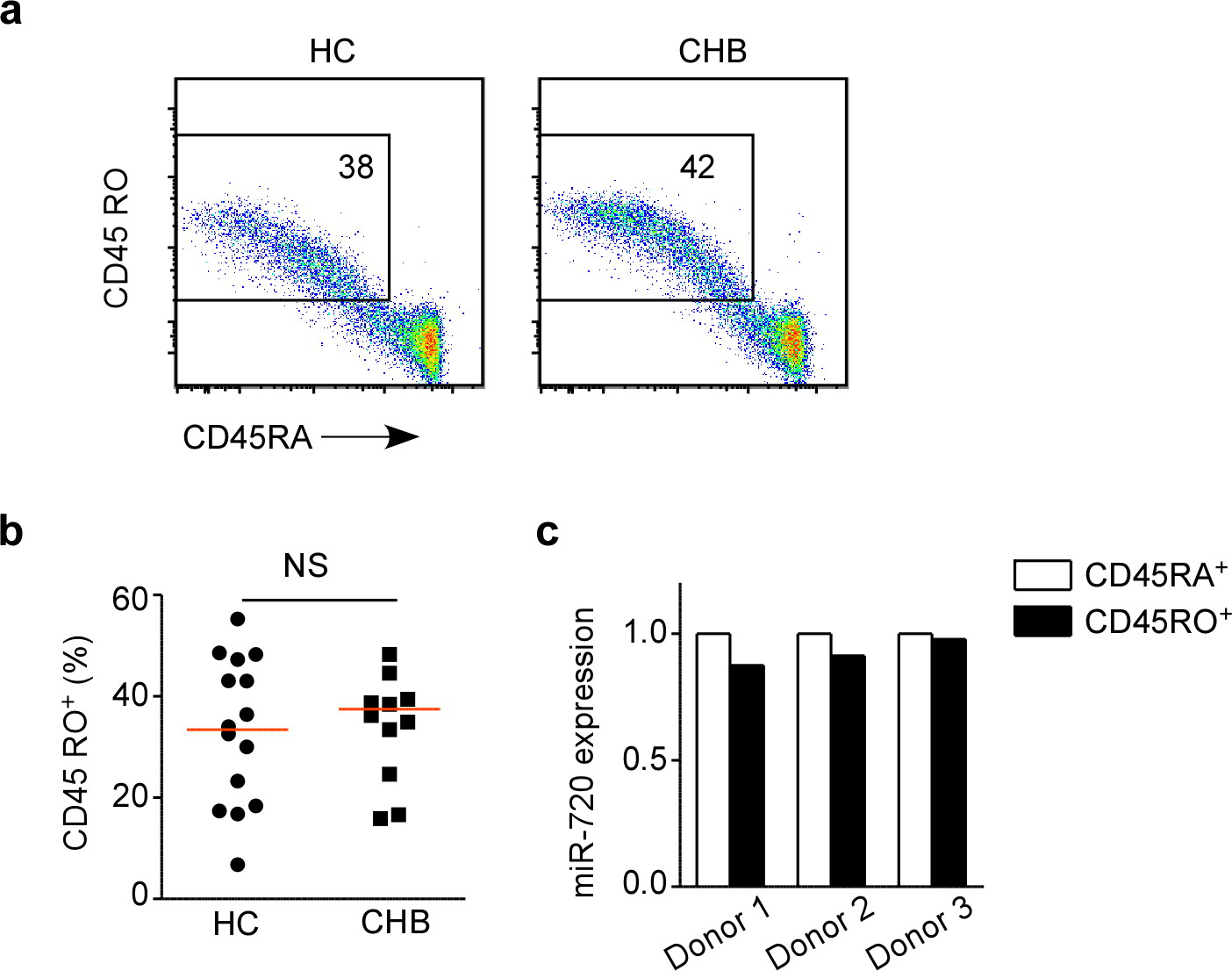


**Figure S3** | **miR-720 expression levels in naïve and memory CD8^+^ T cells from healthy donors**. (a) Flow cytometric analysis of naïve and memory CD8^+^ populations in healthy controls (HC) and CHB patients. Shown are representative FACS profiles of the data in (b). (b) Percentages of naïve and memory CD8^+^ T cells in the healthy controls and CHB patients (HC, n=15; CHB, n=12; NS, not significant). *t*-test. (c) Relative expression of miR-720 in naïve and memory CD8^+^ T cells from healthy donors as determined by qPCR.


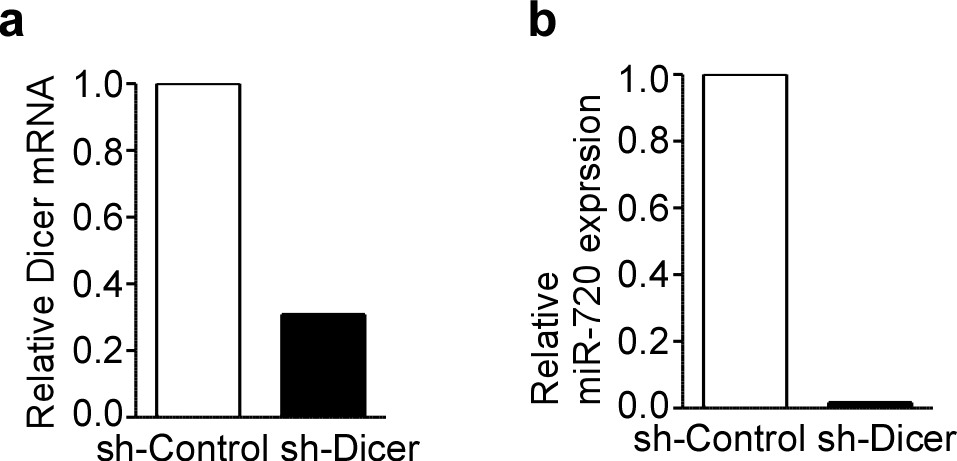


**Figure S4 |** **miR-720 is a Dicer dependent microRNA**. (a) Dicer expression was decreased in shRNA Dicer knockdown 293T cells compared with that from shRNA control. (b) miR-720 expression level in the Dicer knockdown 293T cells.


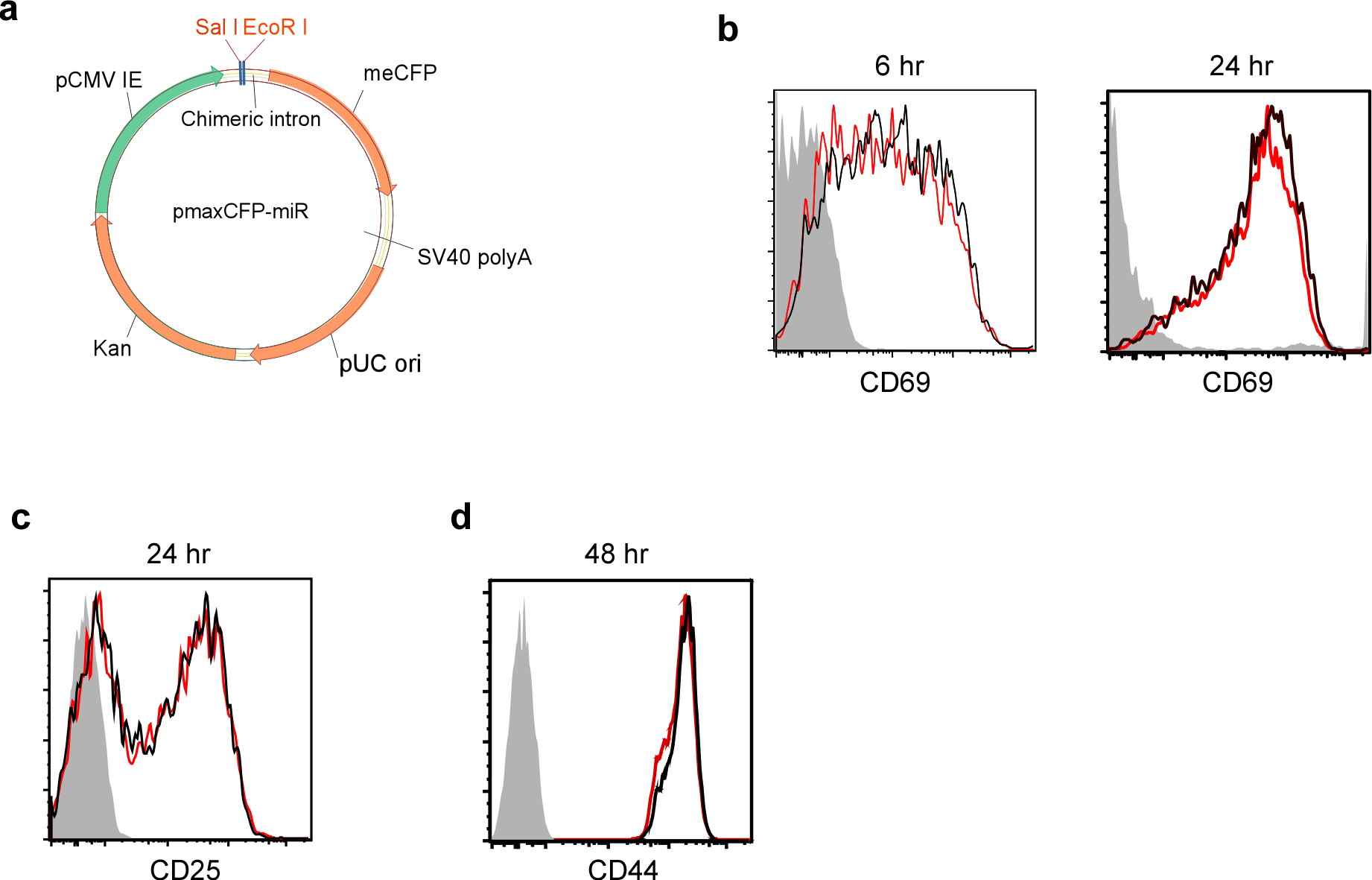


**Figure S5 |** **Regulation of T cell proliferation by miR-720**. (a) Schematic map of microRNA expression vector. (b-d) CD69, CD25, and CD44 expression in miR-720-overexpressing CD8^+^ T cells after TCR stimulation at the indicated time points. Solid grey, black, and red lines represent isotype control, pmax-CFP-miR, and pmax-CFP-miR-720, respectively.


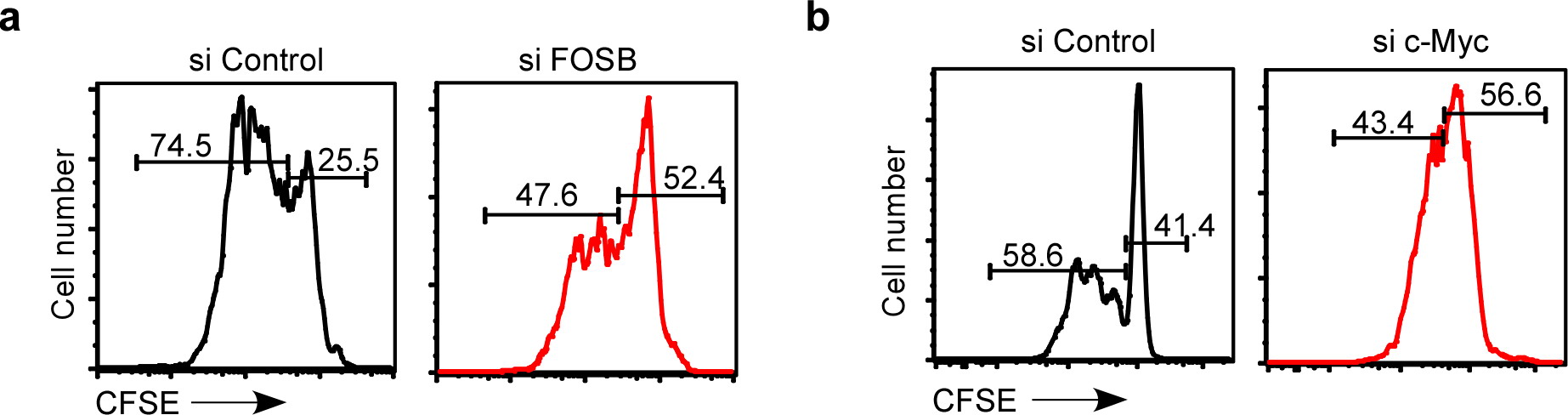


**Figure S6 | Cell cycle genes as targets of miR-720**. (a) Proliferation of FOSB-silenced CD4^+^ T cells. (b) Proliferation of c-Myc-silenced CD4^+^ T cells.


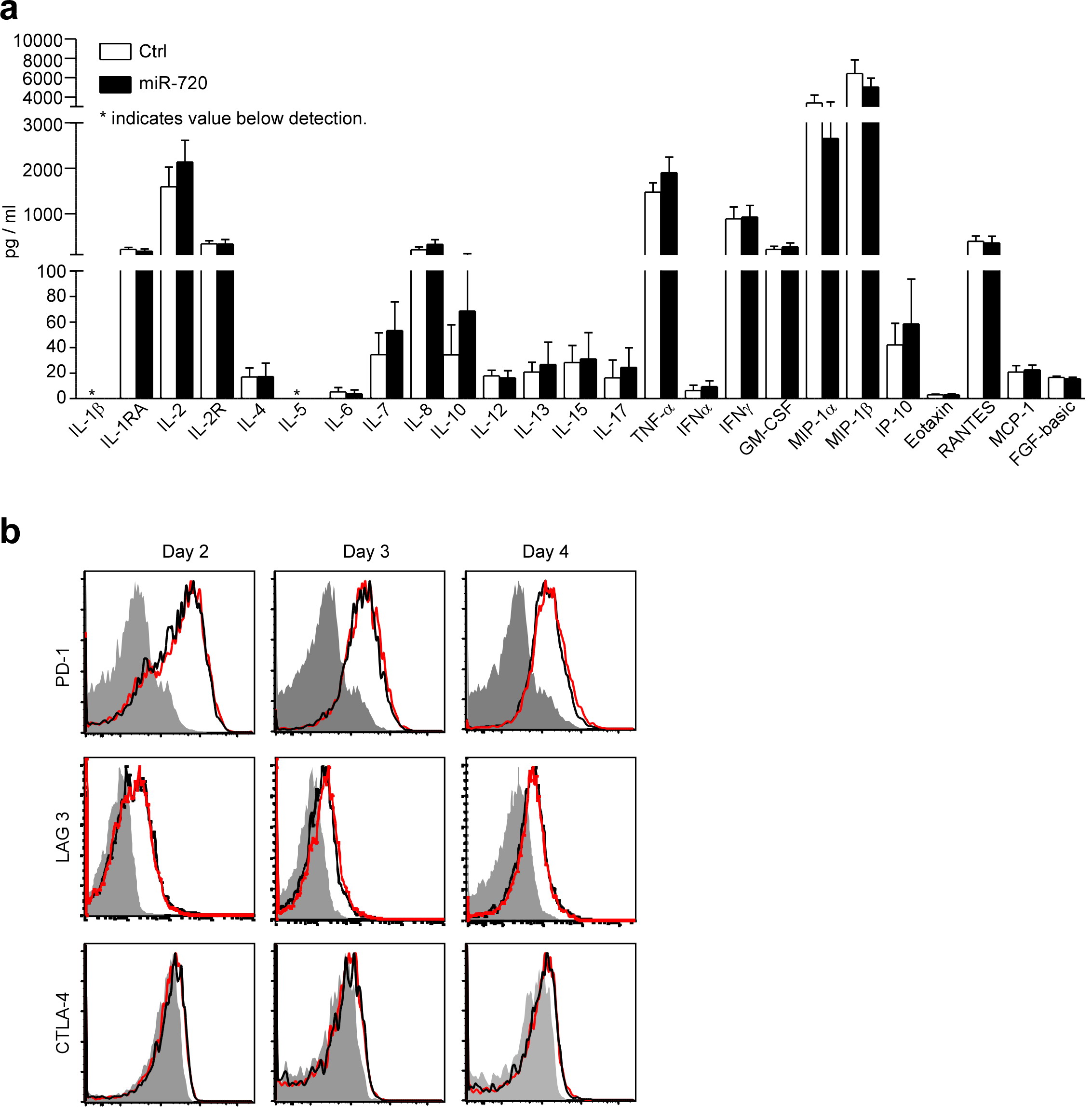


**Figure S7 | Cytokine profile of miR-720 overexpressing CD8^+^ T cells**. **(**a**)** miR-720 was overexpressed in human primary CD8 T cells using pmax-GFP vector. The GFP positive CD8 T cells were sorted by flowcytometry and activated by CD3 and CD28 antibodies for 24hrs, then stimulated with PMA and ionomycin for 6hrs. The cell free supernatant was harvested for lunimex assay. (b) miR-720 was overexpressed in human primary CD8 T cells using pmax-GFP vector. The inhibitory receptors PD-1, LAG3 and CTLA-4 were analyzed using flowcytometry at indicated time point after CD3 and CD28 antibodies stimulation. Solid gray, black line and red line indicate isotype control, Control GFP and miR-720-GFP respectively.

**Table S1. Clinical data of healthy donors and CHB patients.**

| **Parameters** | **CHB** | **Healthy Controls** |  |
| --- | --- | --- | --- |
| n | 45 | 24 |  |
| Age (years), median (range) | 34 (19-61) | 29 (22-48) |  |
| Sex (M:F) | 3:2 | 1:1 |  |
| Serum HBV DNA (IU/ml) (range) | 3.57×10^4^-4.46×10^8^ | 0 |  |
| Serum total bilirubin (μmol /L), median (range) | 49.6 (4.7 - 203) | --- |  |
| AST (IU/L), median (range) | 238 (24 - 703) | --- |  |
| ALT (IU/L), median (range) | 377 (33.5 - 1233) | --- |  |
